# The ongoing legacy of Indigenous family separation: Long-term outcomes of child welfare involvement among American Indian and First Nations youth

**DOI:** 10.1016/j.chiabu.2026.107884

**Published:** 2026-01-12

**Authors:** Stefanie Gillson, Dane Hautala, Rachel Steinberg, Melissa Walls

**Affiliations:** a Yale School of Medicine, Child Study Center, 333 Cedar St, New Haven, CT, 06510, United States of America; b Johns Hopkins Center for Indigenous Health, 1915 South St, Duluth, MN, 55812, United States of America

**Keywords:** Indigenous, First nations, Native Americans, Family separation, Child welfare, Foster care, Psychosocial outcomes, Community-based research, American Indian

## Abstract

**Background:**

Despite policies designed to preserve family structures, Indigenous children in the U.S. and Canada remain disproportionately removed from their homes and communities. This systemic removal represents an ongoing form of historical trauma with intergenerational consequences, yet research on its long-term psychosocial effects remains limited.

**Objectives:**

To examine the prevalence of foster care and adoption among a sample of Indigenous youth and their associations with psychosocial outcomes in early adulthood.

**Participants and setting:**

Data come from a community-based participatory longitudinal study of Indigenous families from eight reservations and reserves in the U.S. and Canada (*N* = 708), collected between 2002 and 2020.

**Methods:**

Multivariate regression models assessed associations between any child welfare involvement during childhood/adolescence (i.e., foster care and/or adoption) and young adult family (i.e., family satisfaction and cohesion) and psychosocial (i.e., flourishing, depressive symptoms, and marijuana use) outcomes.

**Results:**

Overall, 16.73 % of participants experienced child welfare placement during childhood or adolescence. Compared with Indigenous youth who were never placed in child welfare, those who experienced placement had lower odds of family satisfaction (*OR* = 0.40; *p* < .05), reported decreased family cohesion (*b* = −1.38; *p* < .05), lower rates of flourishing (*b* = −1.44; *p* < .05). Despite showing significant bivariate associations, child welfare placement was not associated with depressive symptoms (*b* = 0.20; *p* = .15), and only marginally associated with marijuana use (*b* = 0.59; *p* = .06).

**Conclusions:**

Indigenous family involvement in the child welfare system remains disproportionately high and is associated with adverse psychosocial outcomes in adulthood. Findings underscore the need for systemic reforms and culturally responsive, community-driven interventions to support Indigenous families.

North American Indigenous (i.e., American Indian and Alaska Native [AIAN], Canadian First Nations) families and communities have endured centuries of forced family separations due to colonization policies designed to dismantle Indigenous kinship systems. The significant and ongoing consequences of these unresolved historical traumas are evident in the overrepresentation of Indigenous youth in the child welfare system (Caldwell & Sinha, 2020; U.S. Department of Health and Human Services, 2023). Policies such as the Indian Child Welfare Act (ICWA) in the United States (U.S.) and Bill C-92 in Canada aim to minimize the removal of Indigenous children and uphold Tribal and First Nations sovereignty. However, Indigenous children continue to be displaced from their families at disproportionately high rates (Hahmann et al., 2024; U.S. Department of Health and Human Services, 2023), perpetuating the legacy of historical trauma by disconnecting children from communities and culture (Indian Health Service [IHS], 2019; Kirmayer et al., 2014).

Historical trauma (HT), which encompasses the cumulative effects of colonization trauma, marginalization, and oppression across generations, has been associated with increased feelings of isolation, breakdowns in community and kinship ties, higher rates of mental health burdens, and substance use (Evans-Campbell, 2008; Hatala et al., 2019; Smallwood et al., 2021). One of the most damaging colonial policies was the forced removal of Indigenous children through boarding schools in the U.S. and residential schools in Canada, existing from the late 19th to mid-20th centuries with the mission to “Kill the Indian, Save the Man” (Lomawaima & Ostler, 2018). Indigenous children were forcibly removed from their homes, forbidden to speak their traditional languages (Adams, 1995) or practice their cultural teachings (Duran & Duran, 1995), and subjected to settler-colonial values and Christian religiosity (Glenn, 2015). Canada has formally acknowledged these schools as enactors of cultural genocide (Truth and Reconciliation Commission of Canada, 2015). Although the U.S. has not made a similar acknowledgement, Interior Secretary Deb Haaland launched the Federal Indian Boarding School Initiative to address the intergenerational impact of boarding schools. In 2024, President Biden also formally apologized for the U.S. government’s role in the system (Haaland, 2025).

While boarding and residential schools began closing in the 1960s through the 1980s, policies in both the U.S. and Canada continued to promote the removal and adoption of Indigenous children by white families (McKenzie et al., 2016; Royal Commission on Aboriginal Peoples, 1996; Thibeault & Spencer, 2019). These policies justified forced removals by framing Indigenous families as neglectful due to poverty and lack of understanding of traditional kinship structures (Jacobs, 2013). Historically, many Indigenous children were raised collectively by parents, grandparents, extended family, and community members, in contrast to Western ideals of family rooted in nuclear structures (Gray et al., 2008). By 1978, as many as 35 % of Indigenous children in the U.S. were placed in substitute care, with 85 % placed in non-Native homes (Snowshoe et al., 2017). This period is commonly referred to as the “Sixties Scoop” in Canada, during which similar rates of child removals occurred without the consent or knowledge of Indigenous families (Johnston, 1983, p. 23).

Indigenous resistance to these practices brought significant reforms, including the passage of ICWA in 1978 in the U.S., which upheld Tribal sovereignty over their children and recognized the importance of keeping them within their community. ICWA mandates that states notify Tribes when an AIAN child is removed from their home due to safety concerns, allowing them to intervene and transfer oversight to Tribal governance. It also requires prioritizing placements with extended family or AIAN foster homes (ICWA, P.L. 95–608). Over decades of implementation, the elements of ICWA have remained consistent, but the interpretation has changed over time. The United States Supreme Court has heard multiple ICWA cases, the most recent being Haaland v. Brackeen (2023). The Court has not overturned ICWA in any of these cases, but previous cases (Adoptive Couple v. Baby Girl, 2013) have led to new binding federal guidelines. This includes holding agencies accountable to determine whether ICWA applies to any child and notifying appropriate parties, such as the Tribe (Bureau of Indian Affairs, 2016). Despite these victories, ICWA has faced challenges related to accessibility, inadequate training, poor compliance, and insufficient funding for both federal oversight and implementation resources for Tribes (Haight et al., 2018; ICWA Compliance Task Force, 2017; Wahl, 2010).

In Canada, recent federal legislation, Bill C-92 (2019), opens the possibility for the recognition of Indigenous self-determination in child welfare. The bill aims to address the historical injustices regarding forced removals and lead to shifts in policy and practice in Indigenous child welfare. Although both ICWA and Bill C-92 share a similar purpose of preserving Indigenous families, there are distinct differences. While ICWA gives jurisdiction to tribal courts to oversee child welfare proceedings, Canada lacks a Tribal court system. Additionally, there is no federalized child protection system in Canada; instead, data is decentralized across provinces, leading to fragmented knowledge (Sinha et al., 2021). Drawing from decades of ICWA implementation, Hahn, Caldwell and Sinha (2020) highlight lessons that could support a more effective implementation of Bill C-92, such as the need for strong notification mechanisms, formal recognition of Indigenous jurisdiction, compliance monitoring, and intergovernmental coordination. Like ICWA, Bill C-92 encountered legal challenges shortly after its passage, ultimately leading to its affirmation by the Supreme Court of Canada in 2024.

Despite these legal efforts, Indigenous children continue to experience disproportionate involvement in the child welfare system. In the U.S., they represent 2 % of the foster care population despite comprising only 1 % of the total child population (U.S. Department of Health and Human Services, 2023) and have the highest rates of out-of-home care among all racial and ethnic groups (Newman & Fort, 2017). In Canada, this disparity is even more pronounced, with Indigenous children accounting for over half of the foster care population in 2021, despite representing just 7.7 % of the child population aged 0 to15 (Hahmann et al., 2024; Statistics Canada, 2022). This overrepresentation has continued to increase, even following the passage of Bill C-92 in 2019. For example, Indigenous children comprised 47.8 % of the foster care population in 2011 and 53.8 % by 2021 (Statistics Canada, 2022). Indigenous children are also more likely to remain in the child welfare system longer and experience a greater number of placements compared to non-Indigenous children (Harris, 2021; Sinha et al., 2021).

These persistent disparities are part of a larger pattern of systemic removal of Indigenous children that has disrupted traditional kinship systems by weakening intergenerational ties and limiting exposure to Indigenous parenting role models (Bussey & Lucero, 2013). Although kinship care is seen as a protective factor and should be prioritized, studies examining placement outcomes for Indigenous children have produced mixed results that vary by state and province (Barth et al., 2002; Fallon et al., 2021; Carter, 2009; Francis, Hall, et al., 2023). As Evans-Campbell (2008) notes, governments have not historically considered Indigenous families as an appropriate place to raise children, a belief that can become internalized by parents and children. As such, high rates of child welfare involvement are hypothesized to have intergenerational consequences in the form of decreased family cohesion and satisfaction in adulthood.

The linkages between HT and poorer health among Indigenous populations are theoretically and empirically supported by models such as the Indigenist stress-coping model (Walters & Simoni, 2002). This model posits that many health problems experienced by Indigenous people are directly connected to colonization and its associated forms of discrimination, while also advocating for empowerment and sovereignty. Grounded in historical trauma, this model aligns with life course frameworks, particularly in its conceptualization of culturally relevant stressors. In this study, child welfare placement is examined as one such stressor. While the effects of historical family separations, such as boarding schools, are well-documented among Indigenous populations–including enduring impacts on mental health, substance use, and subsequent generations (Bombay et al., 2011; Elias et al., 2012; Walls & Whitbeck, 2012)–the consequences of contemporary family separation remain less clear. Existing research on Indigenous individuals has primarily focused on their interactions with the child welfare system or the implementation of policies like ICWA (Francis et al., 2023; Haight et al., 2018). Several studies focus on health and wellbeing while in foster care (Landers et al., 2021), whereas research on adult outcomes has largely focused on reunification (Landers et al., 2017; Landers et al., 2021). Regardless of context, family separation can lead to profound psychological distress, impaired social and emotional development, and increased vulnerability to stress-related disorders (National Academies of Sciences, Engineering, and Medicine, 2016) (Canadian council on Social Development). These harms may be compounded for Indigenous individuals given the broader context of colonization and its enduring impacts. Thus, family separation during childhood and adolescence can be conceptualized as a major form of trauma that reverberates across the life course. Consequently, involvement in the child welfare system is hypothesized to decrease psychosocial well-being and increase depressive symptoms and substance use in early adulthood.

Given the persistent inequities in child welfare placements and the limited understanding of their long-term impacts on family dynamics and mental well-being, there is an urgent need for research examining the effects of contemporary separations among Indigenous children. This study aims to quantify the prevalence of child welfare involvement during childhood and adolescence among a large longitudinal sample of Indigenous youth in the U.S. and Canada. Additionally, it investigates the associations between experience with the child welfare system and measures of psychosocial well-being and family outcomes in early adulthood. We hypothesize that youth who have been placed in the child welfare system will have lower family satisfaction and cohesion in their current family environment and decreased psychosocial well-being compared to youth who have never been removed from their homes.

## 1. Method

### 1.1. Sample

Data for these analyses come from Healing Pathways (HP), a longitudinal community-based participatory research study of Indigenous families in the Great Lakes region of the U.S. and Canada. HP is a partnership between university researchers and eight reservation/reserve communities. The data come from one Indigenous cultural group in the Upper Midwest of the U.S. and several bordering Canadian First Nations reserves. Indigenous youth and their caregivers who lived on or within 50 miles of their respective reservation/reserve were enrolled at baseline. As such, these data may not be generalizable to other Indigenous cultural groups in the United States and Canada. Community Research Councils (CRCs) at each site worked with the university team to choose and adapt survey measures, pilot test survey instruments, hire and supervise local interviewers, recruit participants, gather data, and contextualize, interpret, and disseminate study findings. University researchers, including an Indigenous PI, provided the community-based interviewers with intensive multi-day training in research ethics and survey research, followed by structured individual practice and continuous team support throughout the data collection process. Survey interview data were collected via paper and pencil. All study procedures have been approved by the University of Nebraska, the University of Minnesota Institutional Review Boards, and CRCs in all partner communities

To date, HP includes 11 waves of data collected over two decades and is ongoing. Target participants (Generation 2, or G2) were initially recruited using tribal enrollment rosters of age-eligible children who were enrolled citizens living on or near (i.e., within 50 miles) reservations or reserves of partner communities. The research team successfully recruited 735 children at baseline, and the recruitment rate was 79.4 %. A total of 27 participants passed away by Wave 9 and were removed from the sample (analytic sample *n* = 708). Beginning in 2002, trained community members conducted annual interviews with target adolescent participants (G2) and at least one caregiver (Generation 1; G1) over eight years (Waves 1–8; mean youth age at baseline = 11.1 years). Starting in 2017, target (G2) participants were interviewed again in early adulthood (mean age at Wave 9 = 26.3 years) for three additional years. More information about the HP study design can be found in Walls et al., 2021 and Whitbeck et al., 2014.

### 1.2. Measures

In Waves 2–7, interviewers asked caregivers (G1), “During the past year, has [name of child] lived with this family all of the time, or split time between two or more living situations?” Caregivers who responded that the target participant (G2) had lived elsewhere were then asked, “Where did [name of child] live?” and interviewers recorded their verbatim response. At Wave 1, caregivers (G1) were asked whether there has ever been a time of one month or longer when the target adolescent (G2) did not live with them. For those who responded yes, a subsequent question asked where the target adolescent lived and for how long. We coded these qualitative responses as child welfare placement (i.e., foster care and adoption) and other (e.g., living with another family member, juvenile justice system). An overall measure of any child welfare involvement during childhood and adolescence was created by comparing any foster care and adoption with all other categories (0 = no child welfare involvement; 1 = any child welfare involvement).

Two family-related outcomes were assessed in young adulthood. First, *family satisfaction* is a one-item measure adapted from Schumm et al. (1986). Participants were asked, “how satisfied are you with your relationship with your family?” Response options were (0) very dissatisfied, (1) somewhat dissatisfied, (2) neither dissatisfied nor satisfied, (3) somewhat satisfied, (4) very satisfied. Second, *family cohesion* was measured using a five-item scale adapted from the Moos Family Environment Scale (Moos & Moos, 2013). Participants were asked whether they agreed or disagreed with statements such as, “you are proud to be part of your family.” Response options were (0) strongly disagree, (1) disagree, (2) neither agree nor disagree, (3) agree, and (4) strongly agree. Items were summed to create a scale of family cohesion (α = 0.89).

Three psychosocial health indicators were examined in young adulthood. *Flourishing* is an eight-item scale adapted from Diener et al. (2010). Respondents were asked whether they agree or disagree with statements such as “I lead a purposeful and meaningful life” and “I am optimistic about our future.” Response options were (0) strongly disagree, (1) disagree, (2) neither agree nor disagree, (3) agree, and (4) strongly agree. Items were summed to create a scale of flourishing (α = 0.89). *Depressive symptoms* were assessed using a nine-item abbreviated version of the Centers for Epidemiological Studies Depression (CES–D) scale (Radloff, 1977). Participants were asked how many days in the past week they experienced various depressive symptoms (e.g., “you felt sad”). Response options were (0) 0 days, (1) 1–2 days, (2) 3–4 days, and (3) 5–7 days. All positively worded items (e.g., “you enjoyed life”) were reverse-coded. Items were summed to create a scale of depressive symptoms (α = 0.88). *Marijuana use frequency* was assessed by asking participants if they had ever used marijuana in the past 12 months. If so, a follow-up question was asked about how often in the past 12 months they had used marijuana. Response options were (0) none, (1) 1 or 2 times, (2) less than once a month, (3) once a month (4) every week, (5) nearly every day, (6) every day. Respondents who reported no past year marijuana use were coded as 0. Marijuana was chosen over other substances (e.g., alcohol) because of its relatively high prevalence rate and variability in use compared to other substances (e.g., alcohol, opioids, stimulants).

Several demographic and relevant concurrent risk and protective factors were included as control variables. *Gender* (0 = male; 1 = female), *residing on* vs. *off reservation/reserve land at baseline* (0 = off reservation/reserve; 1 = on reservation/reserve), *adult personal income* (continuous), and *adult relationship status* (0 = single; 1 = married or in a relationship; 2 = divorced, separated, widowed, and other) were included as demographic covariates. We also controlled for two concurrent risk and protective factors during adolescence that may confound the association between child welfare placement and young adult outcomes. Benevolent childhood experiences (BCEs) were measured using an adapted version of the scale created by Narayan et al. (2018). Respondents were asked whether during their first 18 years of life they had experience multiple positive events (e.g., “did you have at least one caregiver who you felt safe with” and “did you have at least one teacher who cared about you”). Response options were (0) no and (1) yes. Items were summed to create a count of BCEs (α = 0.49). Adverse childhood experiences (ACEs) were assessed using a measure adapted from the Behavioral Risk Factor Surveillance System (BRFSS) study. Respondents were asked whether they experienced 11 forms of childhood trauma before the age of 18 (e.g., sexual and physical abuse, neglect, etc.). Response options were (0) no and (1) yes. Items were summed to create a count of ACEs (α = 0.84).

### 1.3. Analytic strategy

To examine the association between child welfare placement during adolescence and young adult outcomes, multivariate regression models were estimated. Except for family satisfaction, linear regression models with robust standard errors were estimated. Family satisfaction was analyzed using ordinal regression. All covariates were included simultaneously to examine whether child welfare placement explained a significant proportion of variance in the outcomes once relevant adolescent and adult covariates were controlled for. To account for missing data on the covariates and outcomes, multiple imputation by chained equations (MICE) was used in Stata (StataCorp, 2023). All variables included in the analyses were entered into the imputation model, and the study site was added as an auxiliary variable. A total of 50 data replicates were created, and results were pooled across these data replicates using Rubin’s rules (Rubin, 1987).

## 2. Results

### 2.1. Prevalence of child welfare involvement

Using the non-imputed data (see Table 1), 16.73 % of the sample experienced child welfare placement at least once during childhood/adolescence. Among participants who reported being placed in foster care, most experienced their first episode before the age of 10. Table 1 presents descriptive statistics for the total sample and across categories of child welfare placement. At baseline, the sample was evenly split by males (49.73 %) and females (50.27 %), most participants resided on reservation/reserve land (86.73 %), and the mean per capita family income was $5579.82. Several proportions and means were significantly different across child welfare placement categories. Compared to participants who did not experience child welfare placements, those who did had a lower mean per capita family income (*t* = 2.84; *p* = .00), benevolent child experiences (*t* = 4.69; *p* = .00), family satisfaction (*t* = 5.25; *p* - 0.00), family cohesion (*t* = 4.10; *p* = .00), and flourishing (*t* = 4.10; *p* = .00). Moreover, compared to participants who did not experience child welfare placement, those who did reported higher levels of depressive symptoms (*t* = −2.74; *p* = .01) and marijuana use frequency (*t* = −2.97; *p* = .00).

### 2.2. Early adult outcomes in child welfare involvement

Table 2 presents the regression models predicting family-related and psychosocial outcomes. For the two family-related outcomes, even after controlling for demographics and contemporaneous risk/protective factors, participants who had ever experienced child welfare placement during childhood/adolescence had decreased odds of family satisfaction (*OR* = *0.40; p* < *.01*) and lower rates of family cohesion in early adulthood (*b* = −1.38; *p* < .05). For both outcomes, BCEs increased family satisfaction and cohesion, while ACEs decreased them.

For the three psychosocial outcomes, even after controlling for demographics and contemporaneous risk/protective factors, participants who had ever experienced child welfare placement had decreased rates of flourishing (*b* = 1.14; *p* < .05). Despite showing significant bivariate differences, child welfare placement was not associated with depressive symptoms (*b* = 0.20; *p* = .15), and only marginally associated with marijuana use frequency (*b* = 0.59; *p* = .06). ACEs were negatively associated with flourishing (*b* = −0.17; *p* < .05) and positively associated with more frequent marijuana use (*b* = 0.16; *p* < .001). BCEs were positively associated with flourishing (*b* = 1.19; *p* < .001) and negatively associated with depressive symptoms (*b* = −0.17; *p* < .001) and marijuana use frequency (*b* = −0.21; *p* < .05)

## 3. Discussion

This study examined child welfare involvement and its consequences on psychosocial wellbeing during early adulthood among a large longitudinal study of American Indian and First Nation youth. Policies such as ICWA in the U.S. and Bill C-92 in Canada were enacted in response to decades of colonial practices that systemically removed Indigenous children from their families and communities. Despite these policy reforms, Indigenous children continue to experience disproportionately high rates of family separation. The findings of this study highlight how contemporary family separations are rooted in historical trauma that continues to shape long-term psychosocial wellbeing among Indigenous young adults.

These findings confirm significant disparities in child welfare placement in Indigenous populations and add to prior research that Indigenous youth are more frequently placed in environments disconnected from their cultural communities (Galan et al., 2021; Trocmé et al., 2004), reflecting a continuation of patterns of forced family separations. Our finding that 16.73 % of Healing Pathways participants experienced foster care or adoption is similar in magnitude to Yi et al. (2020), who found that 11.4 % of Indigenous children in the U.S. experienced foster care by age 18, more than double the national average of 5.3 % in 2016. Although our measure includes both foster care and adoption, these comparable rates highlight the ongoing overrepresentation of Indigenous children in the child welfare system. Although Canada lacks a national dataset analogous to that of the U.S. (Afifi, 2011), province-specific studies offer important context. For instance, a longitudinal study in Manitoba, which has a large Indigenous population (Statistics Canada, 2022), found that 27.4 % of First Nations parents had a child placed in out-of-home care at some point over a 20-year period (Kenny et al., 2025). While not limited to experiences before age 18, these findings highlight the extensive and disproportionate involvement of Indigenous families in the child welfare system. In contrast, a study from Quebec, which has different demographics and child welfare policies, found that 3.4 % of all children had experienced child welfare placement by age 17 (Esposito et al., 2023). These provincial differences illustrate the variability of child welfare involvement across jurisdictions rather than a direct equivalence between provinces. Moreover, previous research has shown that Indigenous children in foster care often face greater mental health challenges (Landers et al., 2017) and experience lower rates of family reunification (Landers et al., 2021) than non-Indigenous children.

Our results show that the consequences of child welfare placement extend into adulthood. These placements were significantly associated with adverse psychosocial and family outcomes, even after accounting for adverse and benevolent childhood experiences. Specifically, participants who experienced child welfare placement during adolescence reported lower levels of family satisfaction, family cohesion, and flourishing in early adulthood. Despite showing significant bivariate associations with child welfare involvement, the multivariate results for depressive symptoms and marijuana use frequency were not significant. These findings align with previous research, including systematic reviews and large-scale studies, which have shown that youth in the child welfare system are more likely to have diminished psychosocial well-being (Engler et al., 2022; McKenna et al., 2021; Seker et al., 2022). Our findings also align with research showing that foster care placements among Indigenous individuals were associated with cultural loss in adulthood, and those with histories of both foster care and ancestral boarding school experiences are associated with increased levels of depression, anxiety, and loneliness (Gillson et al., 2022). Notably, these outcomes persisted after controlling for ACEs and BCEs, underscoring how involvement in the child welfare system alone may represent a distinct form of historical trauma and a pathway through which systemic inequities are reproduced.

Despite generations of systemic oppression, Indigenous communities have demonstrated resilience by maintaining kinship networks that offer built-in protections and play a crucial role in family well-being today. For example, Indigenous grandparents are three times more likely to raise their grandchildren compared to grandparents from other racial or ethnic groups (Mutchler et al., 2007). However, the disruptions caused by family separations reverberate into adulthood and ripple across generations, perpetuating cycles of cultural and kin disconnection that contribute to historical trauma. High rates of removal have weakened intergenerational ties, limited exposure to Indigenous parenting models (Bussey & Lucero, 2013), and may lead caregivers to internalize the colonial message that Indigenous families are unfit to raise their children (Evans-Campbell, 2008). Socio-cultural connectedness, including cultural identity, language, and involvement in traditional and community activities are critical to wellness within Indigenous communities and is associated with positive mental health (Gray & Cote, 2019; Snowshoe et al., 2017; Ullrich, 2019). However, recent research shows that Indigenous children are no more likely to be placed in kinship care (Francis, Hall, et al., 2023), potentially limiting their access to the protective cultural ties.

Systemic reforms are needed to address the overrepresentation of Indigenous children within child welfare services. These deep-rooted disparities reflect systemic racism, often driven by cultural misunderstandings of Indigenous child-rearing practices. Indigenous family systems emphasize relational practices in which young children are embedded within extended family, community, and social networks, rooted through ceremony and culture (Ullrich, 2019; Wesner et al., 2024). This contrasts with Eurocentric models, particularly those based on attachment theory, that dominate child welfare assessments. Such tools are largely focused on the dyad between a parent and child and fail to consider how the meaning of successful parenting varies across cultures (Choate & Lindstrom, 2017). Attachment theory was not intended to be used in child welfare assessments (Choate & Tortorelli, 2022; Forslund et al., 2022) and has limited applicability to Indigenous populations (Choate & Lindstrom, 2017; Neckoway et al., 2007). As a result, Indigenous children are more likely to be removed from their homes due to maltreatment reports, specifically neglect, often tied to structural poverty, inadequate housing, and substance misuse (Haight et al., 2018; Sinha et al., 2013; Sinha et al., 2021). Such removals disrupt traditional kinship systems and contribute to the cycle of historical trauma. Moreover, continued reliance on culturally incongruent assessment tools contributes to the misclassification of Indigenous caregiving as neglectful, reinforcing systemic determinants of health inequities. In response, Indigenous scholars and community leaders have begun developing child development models rooted in Indigenous worldviews (O’Keefe et al., 2022; Wesner et al., 2024).

While policies such as ICWA and Bill C-92 are critical for safeguarding Indigenous families, their inconsistent implementation and ongoing legal challenges hinder meaningful progress. Addressing these disparities requires systemic reforms that prioritize the preservation of Indigenous family structures, implement culturally grounded child welfare practices, and tackle the structural barriers that perpetuate inequities. Despite the harrowing history, Indigenous values of interconnection persist and are critical to overcoming the impacts of colonization (Ullrich, 2019). Prioritizing the role of traditional Indigenous kinship systems is vital for fostering resilience, cultural continuity, and the well-being of Indigenous children and families. Further research is needed to better understand how policy implementation gaps and systemic barriers continue to affect Indigenous families and to identify effective strategies for sustainable change. Together, these findings highlight how child welfare involvement acts as a continuation of historical trauma. Policies aiming to preserve Indigenous families must go beyond legal protections to address the structural inequities and embed Indigenous worldviews into child welfare and public health frameworks to disrupt the cycle of trauma and family separation.

### 3.1. Limitations

The data come from one Indigenous cultural group in the upper Midwest of the U.S. and several bordering Canadian First Nations reserves. Only youth who lived on or within 50 miles of their respective reservation or reserve at baseline were enrolled. Therefore, these data may not be generalizable to other North American Indigenous cultural groups, or Indigenous people who grew up in urban communities. Additionally, the measures and indicators used in this study were not originally designed with child welfare research in mind. For example, responses to the question about where children had lived in the previous year when not with their primary caregiver were open-ended; caregivers often provided brief answers that lacked important contextual details (e.g., court-ordered separations, reasons for separation). Relatedly, these self-report questions may be less accurate as administrative data and may obscure the true prevalence of child welfare involvement in this sample. At the conception of this study, youth who were already in the child welfare system often were not ineligible for enrollment. Consequently, the estimates presented in this study may be conservative. Finally, we are unable to fully leverage the longitudinal structure of the adolescent waves to assess whether timing and duration of child welfare involvement affected young adult outcomes.

## 4. Conclusions

This study highlights the persistent inequities in child welfare involvement and their long-term impacts on the psychosocial well-being of Indigenous individuals. Using community-based longitudinal data, this research contributes to the limited literature on the adult outcomes of contemporary foster care and adoption, emphasizing that these experiences are associated with adverse mental health and family dynamics in adulthood. Specifically, individuals who experienced child welfare placement reported lower family satisfaction, family cohesion, and flourishing. These findings underscore the enduring effects of childhood separations on family and community ties, reaffirming the critical need for culturally responsive policies and interventions that address the unique historical and cultural contexts of Indigenous Peoples. Moving forward, greater efforts are needed to ensure that policies are not only upheld but also implemented with adequate resources, training, and oversight to promote family preservation and cultural continuity, thereby breaking cycles of historical trauma and fostering long-term well-being within Indigenous communities.

## Figures and Tables

**Table 1:** Descriptive Statistics for Total Sample and Child Welfare Placement Categories.

	Total Sample %/Mean (S.D.)	No Child Welfare Placement %/Mean (S.D.)	Foster Care/Adoption %/Mean (S.D.)	Test-Statistic

Gender				
Male	49.73 %	49.07 %	45.35 %	*χ*^2^ = 0.40; *p* = .53
Female	50.27 %	50.93 %	54.65 %	
Location				
Off reservation/reserve	13.27 %	11.21 %	14.12 %	*χ*^2^ = 0.58; *p* = .45
On reservation/reserve	86.73 %	88.79 %	88.79 %	
Benevolent childhood experiences*	7.31 (1.03)	7.47 (0.90)	6.85 (1.33)	*t* = 4.69; *p* = .00
Adverse childhood experiences	3.73 (3.07)	3.46 (2.97)	4.20 (3.50)	*t* = −1.79; *p* = .07
Baseline per capita family income*	$5579.82 ($4933.02)	$5686.33 ($5256.43)	$3967.03 ($2973.95)	*t* = 2.84; *p* = .00
Adult relationship status				
Single	41.94 %	42.70 %	46.48 %	*χ*^2^ = 1.97; *p* = .37
Married or living with partner	56.07 %	54.80 %	53.52 %	
Other	1.99 %	2.49 %	0.00 %	
Family Satisfaction*	3.17 (1.11)	3.31 (0.97)	2.56 (1.38)	*t* = 5.25; *p* - 0.00
Family Cohesion*	14.44 (4.18)	14.87 (3.89)	12.65 (4.79)	*t* = 4.10; *p* = .00
Flourishing*	24.00 (4.55)	24.51 (4.40)	22.09 (4.52)	*t* = 4.10; *p* = .00
Depressive Symptoms*	0.86 (1.02)	0.79 (1.01)	1.17 (1.11)	*t* = −2.74; *p* = .01
Marijuana Use Frequency*	1.63 (2.13)	1.54 (2.12)	2.38 (2.19)	*t* = −2.97; *p* = .00

* *p* < .05.

**Table 2:** Regression Models Predicting Young Adult Family and Psychosocial Outcomes (*N* = 708).

	Family Satisfaction	Family Cohesion	Flourishing	Depressive Symptoms	Marijuana Use Frequency
					
	*OR* (95 % CI)	*b* (95 % CI)	*b* (95 % CI)	*b* (95 % CI)	*b* (95 % CI)

Adolescent child welfare placement	0.40** (0.23, 0.70)	−1.38* (−2.51, −0.25)	−1.45* (−2.57, −0.32)	0.20 (−0.07, 0.47)	0.59 (−0.02, 1.19)
Female	1.01 (0.68, 1.50)	−0.15 (−0.90, 0.60)	0.23 (−0.61, 1.07)	0.05 (−0.15, 0.26)	−0.58** (−0.97, −0.19)
On reservation/reserve	0.83 (0.51, 1.36)	−0.89 (−1.87, 0.09)	−0.78 (−1.90, 0.34)	0.11 (−0.16, 0.37)	0.22 (−0.30, 0.74)
Benevolent childhood experiences	1.63*** (1.36, 1.94)	1.06*** (0.69, 1.43)	1.19** (0.80, 1.58)	−0.17 (−0.27, −0.08)	−0.21* (−0.40, −0.01)
Adverse childhood experiences	0.91* (0.85, 0.98)	−0.20** (−0.33, −0.07)	−0.17* (−0.31, −0.02)	−0.01** (−0.04, 0.03)	0.16*** (0.10, 0.23)
Per capita family income	0.95*** (0.92, 0.99)	0.03 (−0.04, 0.10)	0.02 (−0.05, 0.10)	−0.01 (−0.04, 0.01)	−0.02 (−0.06, 0.02)
Adult relationship status^a^					
Married or living with partner	1.41 (0.95, 2.09)	0.61 (−0.19, 1.40)	1.51*** (0.67, 2.34)	−0.20* (−0.40, −0.01)	−0.09 (−0.49, 0.31)
Other	0.52 (0.17, 1.62)	−2.45 (−4.95, 0.06)	1.69 (−1.51, 4.89)	−0.59 (−1.33, 0.15)	−0.77 (−2.16, 0.62)

a Reference category – Single.

* *p* < .05.

** *p* < .01.

*** *p* <. 001.

## Data Availability

The data that has been used is confidential.
